# Is there any potential link among caspase-8, p-p38 MAPK and bcl-2 in clear cell renal cell carcinomas? A comparative immunohistochemical analysis with clinical connotations

**DOI:** 10.1186/1746-1596-4-7

**Published:** 2009-02-17

**Authors:** Vassilis Samaras, Maria Tsopanomichalou, Angeliki Stamatelli, Christos Arnaoutoglou, Efstathios Samaras, Marianthi Arnaoutoglou, Hercules Poulias, Calypso Barbatis

**Affiliations:** 1 Department of Pathology, Hellenic Red Cross Hospital, Athens, Greece; 2 1st Department of Pathology, University of Athens Medical School, Athens, Greece; 3 Department of Cytopathology, Evangelismos General Hospital, Athens, Greece; 4 Department of Neurosurgery, Hellenic Red Cross Hospital, Athens, Greece; 5 1st Department of Neurology, Aristotle University of Thessaloniki, Thessaloniki, Greece; 6 Department of Urology, Hellenic Red Cross Hospital, Athens, Greece

## Abstract

**Background:**

Clear cell renal cell carcinomas (ccRCCs) constitute the most common renal carcinomas, characterized by a relatively aggressive clinical course. Thus, scientific research is targeting towards the identification of immunohistochemical and molecular markers that could be useful regarding diagnosis, appropriate therapy and prediction of prognosis. In the present study we assessed and correlated the expression of caspase-8, phosphorylated p38 mitogen-activated protein kinase (p-p38) and bcl-2 protein with histopathological features and clinical outcome of 27 patients with ccRCCs.

**Method:**

Immunohistochemistry in formalin-fixed and paraffin-embedded tissue sections was performed. The associations among various features were assessed utilizing statistical analysis.

**Results:**

We found that increased expression of cytoplasmic caspase-8 and bcl-2 protein was strongly associated with low Fuhrman's grade of carcinomas (p = 0.019 and p = 0.041, respectively). On the other hand, increased p-p38 expression was significantly related to high Fuhrman's grade (p = 0.006). Moreover, high bcl-2 expression was correlated with low pathological stage of ccRCCs (p = 0.026). Increased expression of cytoplasmic caspase-8 as well as low-grade tumors (grade 1 and 2) implied a greater probability of patients' survival, in univariate statistical analysis (p = 0.037 and p = 0.019, respectively). Neither p-p38 nor bcl-2 expression was significantly linked to patients' survival. There were not emerged statistically significant associations among caspase-8, p-p38 kinase and bcl-2 protein.

**Conclusion:**

For the first time the prognostic impact of caspase-8 and p-p38 was studied in a series of ccRCCs, using immunohistochemistry in formalin-fixed and paraffin-embedded tissue sections. The suggestive relationship of caspase-8 with patients' clinical outcome, as well as the role of p-p38 within different grade categories, mandates further studies in larger cohorts of RCCs.

## Background

Conventional (clear) RCCs (ccRCCs) are characterized by their relatively poor prognosis due to late presentation and resistance to various therapeutical manipulations [1]. Thus, the identification of new immunohistochemical and molecular markers that could provide better prognostic information for each patient is needed. In this context, the role of factors, such as bcl-2 protein, caspases and mitogen-activated protein kinases (MAPKs), influencing cell survival-proliferation and apoptosis, has not been fully understood yet, within ccRCCs.

Bcl-2 is an intracellular membrane protein capable of blocking the programmed cell death through regulation of the mitochondrial apoptotic pathway [2,3]. Although bcl-2 is frequently upregulated in RCCs, studies of its exact relationship with patients' clinicopathological features have given controversial results thus far [4-6].

Caspases are proteases characterized by their essential action in the apoptotic pathway [7]. Caspase-8, particularly, plays a decisive role as an initiator caspase in the extrinsic apoptotic pathway since its activation leads to caspase-3 activation and accordingly to apoptosis [7]. However, bibliographical data regarding caspase-8 immunohistochemical expression in RCCs are limited [8-10].

p38, a member of the MAPKs group, participates in a signaling cascade regulating cellular responses to cytokines and stress and seems to play a role in cell proliferation and apoptosis within RCCs [11,12]. Few former studies assessed the immunohistochemical expression of p38 in RCCs but the impact of activated (phosphorylated) p38 (p-p38) on patients' survival has not been described yet [12,13].

Experimental data suggest that p38 pathway may function both upstream and downstream of caspases (including caspase-8) in the apoptotic response [14,15]. Moreover, p38 kinase appears to play a crucial role regarding bcl-2 phosphorylation and its subsequent inactivation, leading to an increased apoptotic effect [16,17]. Nevertheless, this dual, yet controversial, function of p38 kinase which links three different apoptotic pathways has never been studied within RCCs.

The purpose of our study was to assess and compare the immunohistochemical expression of p-p38, bcl-2 and caspase-8 in ccRCCs, identifying any associations with Fuhrman's grade, pathological stage and patients' clinical outcome. To our knowledge this is the first study attempting to explore the specific prognostic influence of p-p38 and caspase-8 immunohistochemical expression within ccRCCs.

## Method

27 patients with ccRCCs [15 men and 12 women, mean age at operation: 58.15 years (range: 42–77)] underwent radical nephrectomy between 1999 and 2003 at our Hospital. Follow-up information was available in all cases, informed consent was obtained from all patients and the study was approved by the scientific committee of the Hospital. The mean time of follow-up was 70.63 months (range: 11–105), during which 4 patients died of the disease [mean survival: 40.75 months (range: 11–54)] whereas 23 were alive [mean follow-up: 75.83 months (range: 44–105)].

All specimens were formalin-fixed and paraffin-embedded. For each patient, one representative block containing more than 80% of neoplastic tissue was histopathologically evaluated. All tumors were graded according to Fuhrman's grading system [18] as: grade 1 (n = 7), grade 2 (n = 13), grade 3 (n = 4) and grade 4 (n = 3). The pathological stage (pT-stage) was determined using the TNM classification of malignant tumors [19] as: pT1a (n = 5), pT1b (n = 6), pT2 (n = 8), pT3a (n = 5) and pT3b (n = 3).

p-p38, bcl-2 and caspase-8 immunostaing was performed in representative sections, cut at 3 μm, using Bond-maX system (Vision BioSystems Ltd, Australia) according to the manufacturer's instructions. The following antibodies were used: anti-phopsho-p38 MAPK (clone 12F8, rabbit mAb, Cell Signaling Technology Inc. USA, diluted: 1/100), anti-bcl-2 protein (clone 124, mouse mAb, Dako Cytomation, Denmark, diluted: 1/50) and anti-caspase-8 (clone 11B6, mouse mAb, Novocastra, UK, diluted: 1/30).

Under light microscope, p-p38 and bcl-2 immunostaining was estimated as the percentage of neoplastic cells with nuclear and cytoplasmic reactivity, respectively, out of the total number of neoplastic cells counted. The same method was also applied for the assessment of cytoplasmic caspase-8 immunostaining. However, because of a marked intratumoral heterogeneity of its expression, a score was given to sections based on the estimated percentage of immunopositive cells (1 = ≤25%, 2 = 26–50%, 3 = 51–75%, 4 = >75%) and on the intensity of the immunostaining (1 = no staining or weak, 2 = moderate, 3 = strong, 4 = very strong). A final combined score of immunostaining was then calculated by adding both scores (weak = score 2–3, moderate = score 4–6, strong = score 7–8). The presence or absence of nuclear caspase-8 staining was also assessed separately and scored as positive or negative (>10% or ≤ 10% positive cells out of the total number of neoplastic cells counted, respectively).

For statistical calculations, p-p38 expression and bcl-2 cytoplasmic expression as well as patients' age were treated as continuous variables. Cytoplasmic and nuclear caspase-8 expression, tumor grade, pT-stage and patients' sex were analyzed as categorical variables. Associations among continuous variables were tested using Spearman's rank correlation coefficient whereas correlations among categorical variables using Chi-square or Fisher's exact test, as appropriate. The relationship between a continuous and a categorical variable was examined using Kruskal-Wallis ANOVA test or Mann-Whitney U-test, as appropriate.

Univariate survival analysis was performed using death due to disease as the endpoint. The effect of the various features on clinical outcome was assessed by plotting survival curves according to Kaplan-Meier method and comparing groups with the Log-rank test. Continuous variables were categorized on the basis of the median value rounded at its nearest 10%. Caspase-8 cytoplasmic expression was treated as a categorical variable (weak versus moderate/strong) as well as patients' gender. Grade and pT-stage were categorized in two groups (grade 1–2 versus grade 3–4 and pT1a-pT1b-pT2 versus pT3a-pT3b, respectively) due to the different biological behavior of these groups within ccRCCs [20,21]. All statistical calculations were completed using the statistical package SPSS, version 13.0, for Windows Software (SPSS, Chicago, Illinois, USA). Differences were considered statistically significant when p-value (two-sided) was < 0.05.

## Results

### Caspase-8, p-p38 and bcl-2 expression

**Figure 1:**
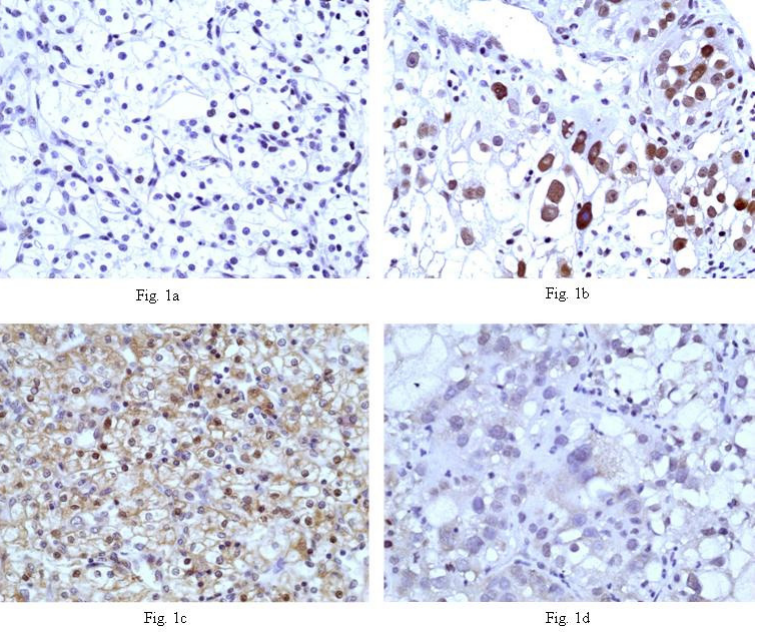
**p-p38 and caspase-8 expression in ccRCC**. **A)**: (p-p38 X400). p-p38 nuclear expression in few malignant cells in a grade 1 ccRCC. **B)**: (p-p38 X400). High p-p38 nuclear expression from the majority of neoplastic cells in a grade 4 ccRCC. **C)**: (caspase-8 X400). Strong cytoplasmic and nuclear expression of caspase-8 in a grade 1 ccRCC. **D)**: (caspase-8 X400). Weak cytoplasmic as well as nuclear expression of caspase-8 in a grade 4 ccRCC. *ccRCC: clear cell renal cell carcinoma*.

Caspase-8 cytoplasmic expression was detected in 27/27 cases [percentage of positive neoplastic cells: 15%–90% (mean value: 48.85%)] [Fig. 1]. Thus, the cytoplasmic score was as follows: score 1 (n = 7 cases), score 2 (n = 8), score 3 (n = 8), score 4 (n = 4). The intensity of cytoplasmic staining was as follows: score 1 (n = 2), score 2 (n = 8), score 3 (n = 9), score 4 (n = 8). Therefore, the final caspase-8 combined cytoplasmic score was: score 1 (weak immunostaining: n = 5), score 2 (moderate immunostaining: n = 13), score 3 (strong immunostaining: n = 9). Concerning nuclear immunoreaction, 18/27 cases were categorized as positive whereas the remaining cases were negative.

p-p38 was detected in 24/27 cases and was exclusively localized in the nuclei of malignant cells [percentage of positive neoplastic cells: 1%–85% (mean value: 19.59%)] [Fig. 1].

**Figure 2:**
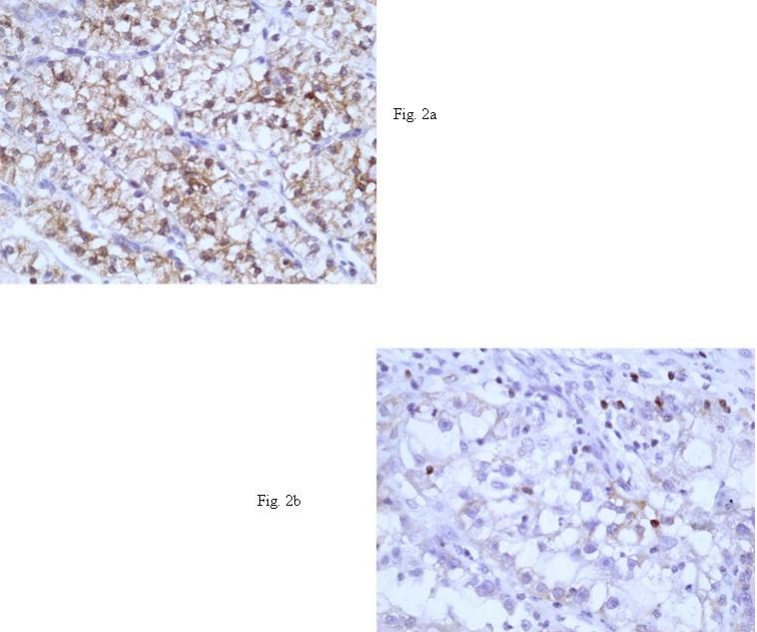
**Bcl-2 expression in ccRCC**. **A)**: (Bcl-2 X400). Strong cytoplasmic expression of bcl-2 protein in a grade 1 ccRCC. **B)**: (Bcl-2 X400). Weak cytoplasmic expression of bcl-2 protein in a few malignant cells in a grade 3 ccRCC. Note also the positive inflammatory cells. *ccRCC: clear cell renal cell carcinoma*.

Bcl-2 cytoplasmic immunoreactivity was detected in 27/27 cases [percentage of positive neoplastic cells: 5%–80% (mean value: 45.19%)] [Fig. 2].

### Correlations among factors and Fuhrman's grade

**Figure 3:**
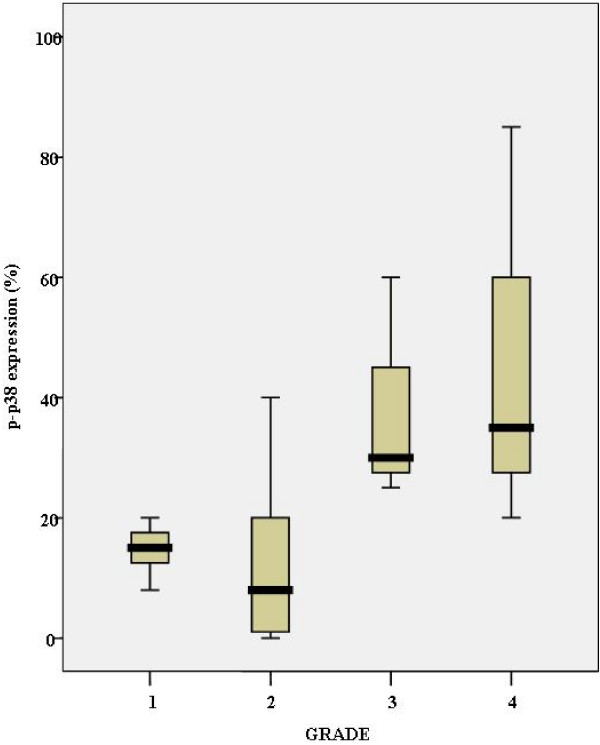
**Schematic illustration of the relationship of p-p38 expression with Fuhrman's grade of 27 clear cell renal cell carcinomas**. p-p38 expression increases with increasing grade in the entire cohort.

In our cohort, cytoplasmic caspase-8 was negatively associated with grade in a statistically significant level [Fisher's Exact Test, p = 0.019]. Nuclear caspase-8 was not connected with grade [Fisher's Exact Test, p = 0.424].

p-p38 expression significantly increased from grade 1 through grade 4 tumors [Kruskal Wallis test, p = 0.006] [Fig. 3].

**Figure 4:**
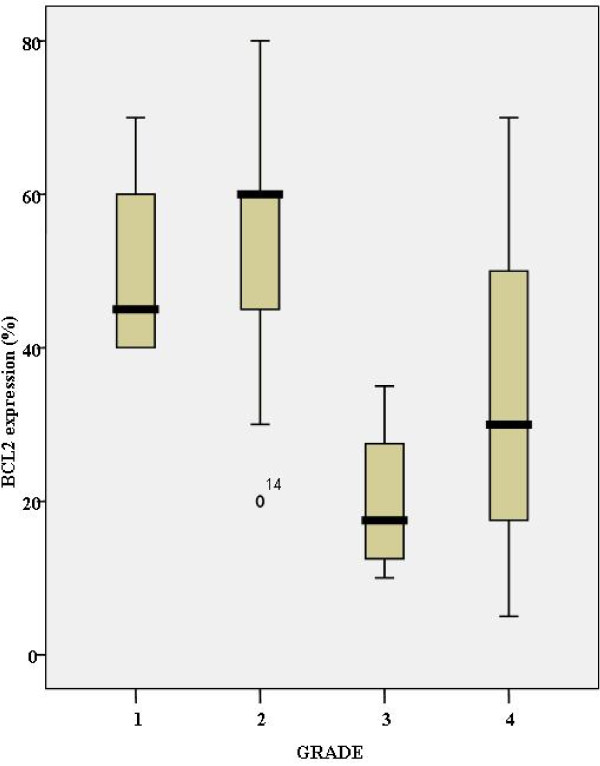
**Schematic illustration of the relationship of bcl-2 expression with Fuhrman's grade of 27 clear cell renal cell carcinomas**. Bcl-2 expression is inversely associated with grade in the entire cohort.

Bcl-2 expression was inversely associated with grade in a statistically significant level [Kruskal Wallis test, p = 0.041] [Fig. 4].

Finally, patients' age and gender were unrelated to grade, in our series [Kruskal Wallis Test, p = 0.601 and Fisher's Exact Test, p = 0.845, respectively].

### Correlations among caspase-8, p-p38, bcl-2 and pT-stage

Bcl-2 expression was inversely associated with pT-stage in the entire cohort [Kruskal Wallis test, p = 0.026].

p-p38 as well as cytoplasmic and nuclear caspase-8 were not significantly associated with pT-stage in our group [Kruskal Wallis test/p = 0.604, Fisher's Exact Test/p = 0.605 and p = 0.302, respectively].

Patients' age and gender as well as grade were not statistically related to pT-stage [Kruskal Wallis Test/p = 0.714, Fisher's Exact Test/p = 0.623, Fisher's Exact Test/p = 0.101, respectively].

### Correlations among caspase-8, p-p38, bcl-2, age and gender

No statistically significant associations were detected among p-p38, caspase-8, bcl-2, patients' age and gender. However, high p-p38 expression tended to be associated with low immunopositivity for cytoplasmic caspase-8 and high immunoreactivity for nuclear caspase-8 [Kruskal Wallis Test, p = 0.202 and p = 0.523, respectively]. p-p38, also, tended to be inversely related to bcl-2 expression [Spearman's rank correlation coefficient, p = 0.433].

### Survival analysis

**Figure 5:**
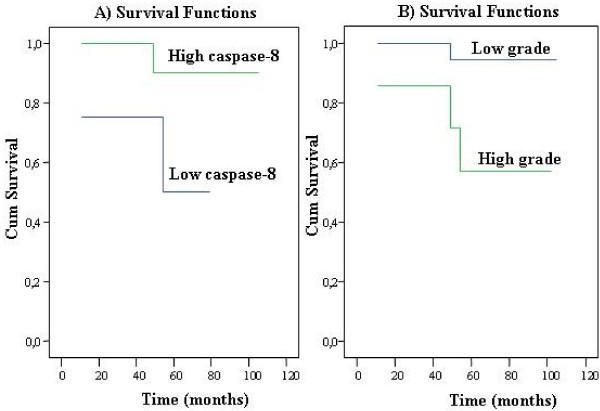
**Kaplan-Meir survival curves in 27 patients with clear cell renal cell carcinomas according to: A) Cytoplasmic caspase-8 expression [green line indicates high expression (moderate/strong) while blue line low expression (weak)]**. **B) **Fuhrman's grade [green line indicates high grade (3 and 4) while blue line low grade (1 and 2)]. According to the statistical analysis, Fuhrman's grading is more significant as a prognosticator than caspase-8 cytoplasmic expression. Cum survival: probability of being a patient alive. Time (months): months since diagnosis.

Univariate analysis demonstrated that higher cytoplasmic caspase-8 [Log Rank Test, weak versus moderate/strong, p = 0.037] as well as lower grade [Log Rank Test, grade 1 and 2 versus grade 3 and 4, p = 0.019] implied a greater probability of survival, in the series of 27 patients [Fig. 5, Table 1].

**Table 1:** The association of studied features with prognosis (univariate survival analysis in the entire cohort)

**Feature**	**P value (Log Rank Test)**
Phospho-p38 expression (<15 vs ≥15)*	0.443
Cytoplasmic caspase-8 expression (weak vs moderate/strong)	**0.037**
Nuclear caspase-8 expression (<10 vs ≥10)	0.257
Bcl-2 expression (<45 vs ≥45)*	0.218
Grade (grade 1/2 vs grade 3/4)	**0.019**
pT-stage (stage pT1a/pT1b/pT2 vs pT3a/pT3b)	0.712
Age (<54 vs ≥54)*	0.273
Gender (male vs female)	0.660

The association of studied features with prognosis was studied with the Log Rank Test. As shown in the table, grade and cytoplasmic caspase-8 expression are both prognosticators, and of these, Fuhrman's grade is more significant. Numbers in bold: statistically significant.

(*): median values.

The mean survival time of patients with high (moderate/strong) cytoplasmic caspase-8 immunoreactivity was 72.77 months whereas the corresponding time for patients with weak caspase-8 cytoplasmic immunoreactivity was 52 months. The mean survival time of patients with low-grade tumors (grade 1 and 2) was 74.40 months while patients with high-grade neoplasms (grade 3 and 4) characterized by a mean survival of 59.86 months.

The remaining features (age, gender, pT-stage, p-p38, nuclear caspase-8, bcl-2) were not significantly related to patients' survival [Table 1].

## Discussion

In the present study, we observed a significant negative association of cytoplasmic caspase-8 with grade of ccRCCs; cytoplasmic caspase-8 was highest in low-grade tumors, in contrast to previous studies in breast and lung carcinomas [22,23]. This negative relationship implies that caspase-8 exerts an apoptotic action within low-grade ccRCCs, hence enhanced cytoplasmic distribution in grade 1 and 2 tumors. Our results seem to reflect the process in which malignant cells of high-grade specimens simultaneously display enhanced proliferative and decreased apoptotic activity [5,24].

A significant association was noted between increased caspase-8 cytoplasmic expression and better patients' survival. This implication has never been reported in RCCs and is in agreement to recently published findings in papillary thyroid carcinomas [25].

p-p38 was detected in the majority of carcinomas localized in the nuclei of the malignant cells, in agreement with a previous study in ccRCCs [12]. Increased p-p38 expression was positively related to high grade ccRCCs, in line with studies performed in a variety of tumors [26,27]. To our knowledge, this is the first study in ccRCCs demonstrating this kind of association.

In contrast to published data regarding the apoptotic and anti-proliferative action of p-p38 in RCCs, our results suggest that p-p38 seems to exhibit a proliferative or anti-apoptotic action, being increased in high-grade tumors [11]. This finding is also strengthened by the identification of decreased expression of the apoptotic cytoplasmic caspase-8 with increasing the degree of malignancy within our group. Indeed, investigators have showed that bone morphogenetic protein 6 played its anti-apoptotic effect in breast cancer through activation of p38 pathway [28].

We additionally detected, in harmony with others, a significant inverse relationship between bcl-2 expression and grade as well as pT-stage of RCCs [6,29]. A possible explanation to this might be based on the suggested antiproliferative role of bcl-2 protein [6,30]. This function, which seems to be distinct from its well-known antiapoptotic action, has been reported in various malignancies [31,32]. Therefore, it could be hypothesized that high expression of bcl-2 prevents cell proliferation, suppresses tumor growth and thereby is associated with a lower grade and pT-stage in RCCs, as previously stated [6].

Provided that a possible but not yet adequately studied mechanism, linking the p38 pathway with caspases activity has been previously implied [14,15], we attempted to correlate the expression of caspase-8 and p-p38 in our series of RCCs. In this framework, researchers have suggested that caspase-8 is essential for the activation of the p38 pathway through death receptors [33], while others pointed out that an enhanced activation of p38 kinase pathway may result in caspase-8 activation and enhancement of apoptosis, based on experiments in a human astrocytoma cell line [34].

However, no statistically significant associations between cytoplasmic or nuclear caspase-8 and p-p38 were revealed in our work, but only a suggestive negative and positive relationship, respectively. These findings backup our aforesaid hypothesis regarding the apoptotic action of cytoplasmic caspase-8 and proliferative or anti-apoptotic role of p-p38 within our ccRCCs [28,35]. Moreover, we cannot exclude a potential inhibitory effect of p38 signaling upon caspase activity, as it has been previously reported [36]. The reason however, for the increased, though statistically insignificant, expression of nuclear caspase-8 in parallel with high p-p38 immunoreactivity, is still to be determined.

As regards p-p38 and bcl-2 association, no strong linkage was identified in our study. However, p-p38 tended to be inversely related to bcl-2 expression. In this context, scientists have stressed that p38 can induce apoptosis via phosphorylation of bcl-2 [16,17]. Concerning RCCs, there are no related studies. According to our work, the negative, though loose, correlation found between p-p38 and bcl-2, constitutes an early indication of a probable antagonistic interaction of these two factors within ccRCCs. The major question to be identified is whether this interaction concerns the proliferative, the apoptotic or both potential functions of these factors.

No statistically important correlation between bcl-2 and caspase-8 was found. This was expected, since these substances act through different apoptotic pathways [3,7]. We also demonstrated that low-grade tumors (grade 1 and 2) depicted greater probability of survival in comparison to high-grade (grade 3 and 4), in accordance with previous data [20,21]. The absence of statistical significance regarding the association of pT-stage with prognosis could be attributed to the small cohort.

## Conclusion

Conclusively, caspase-8 pro-apoptotic function appears to prevail in low-grade ccRCCs and seems to be associated with a better clinical outcome. However, the specific role of cytoplasmic and nuclear caspase-8 needs to be identified. Moreover, p-p38 seems to exert a proliferative rather than an apoptotic role within ccRCCs and is associated with an aggressive phenotype. Finally, the common antiapoptotic role of bcl-2 protein is extremely complicated within ccRCCs and appears to be influenced by other synergic and/or antagonistic pathways. In this regard, p38 MAPK signaling may be of fundamental importance. p38, caspase-8 and bcl-2, though seemingly distinct components of three discrete signaling pathways implicated in cell growth and apoptosis, appear to be characterized, to some extent, by an interrelationship. p38 seems to bridge the above pathways and, in specific tissue substrates, regulates the balance between apoptotic cell death and proliferation. Prospective, larger, experiments are of utmost importance towards the elucidation of the exact clinical role and associations among caspase-8, p-p38 and bcl-2 within the various subtypes as well as the different grade and stage categories of RCCs.

## Competing interests

The authors declare that they have no competing interests.

## Authors' contributions

VS Conception and design of study, writing of manuscript, histological diagnosis. MT  Writing of manuscript, acquisition of clinical data, immunohistochemical staining. AS Writing of manuscript. CA  Analysis and interpretation of statistical data. ES  Writing of manuscript, drafting the manuscript. MA  Analysis and interpretation of statistical data. HP  Revising of manuscript. CB  Revising and editing of manuscript, histological diagnosis. All authors read and approved the final manuscript.
